# Tweet topics and sentiments relating to distance learning among Italian Twitter users

**DOI:** 10.1038/s41598-022-12915-w

**Published:** 2022-06-02

**Authors:** Luisa Stracqualursi, Patrizia Agati

**Affiliations:** Department of Statistics, University of Bologna, 40126 Bologna, Italy

**Keywords:** Psychology and behaviour, Information technology

## Abstract

The outbreak of COVID-19 forced a dramatic shift in education, from in-person learning to an increased use of distance learning over the past 2 years. Opinions and sentiments regarding this switch from traditional to remote classes can be tracked in real time in microblog messages promptly shared by Twitter users, who constitute a large and ever-increasing number of individuals today. Given this framework, the present study aims to investigate sentiments and topics related to distance learning in Italy from March 2020 to November 2021. A two-step sentiment analysis was performed using the VADER model and the *syuzhet* package to understand the overall sentiments and emotions. A dynamic latent Dirichlet allocation model (DLDA) was built to identify commonly discussed topics in tweets and their evolution over time. The results show a modest majority of negative opinions, which shifted over time until the trend reversed. Among the eight emotions of the *syuzhet* package, ‘trust’ was the most positive emotion observed in the tweets, while ‘fear’ and ‘sadness’ were the top negative emotions. Our analysis also identified three topics: (1) requests for support measures for distance learning, (2) concerns about distance learning and its application, and (3) anxiety about the government decrees introducing the red zones and the corresponding restrictions. People’s attitudes changed over time. The concerns about distance learning and its future applications (topic 2) gained importance in the latter stages of 2021, while the first and third topics, which were ranked highly at first, started a steep descent in the last part of the period. The results indicate that even if current distance learning ends, the Italian people are concerned that any new emergency will bring distance learning back into use again.

## Introduction

The COVID-19 pandemic has greatly affected life worldwide. One of the most remarkable effects was the enforcement of social distancing to reduce the spread of the disease. In March 2020^1^, Italy implemented social-distancing measures by enforcing distance learning at all educational stages and online assessments to help continue students’ education^2^. These measures became known as ‘emergency distance learning’ and introduced new experiences and challenges for students, parents, and teachers. In the subsequent months, distance learning gradually moved to ‘integrated digital learning’^3^, which combined remote (virtual classroom) and in-person (traditional classroom) instruction. Unfortunately, this integration was very slow: the reopening of schools has been limited to some Italian regions and has often been only temporary. As post-outbreak *SARS-CoV-2* infections increased, many regions suddenly returned to distance learning for either some grades of school or for all, as happened in Italy’s ‘red zones’.

Social media has been a major and rich data source for research in many domains due to its 3.8 billion active users^4^ across the globe. For instance, researchers analyze user comments extracted from social media platforms (such as Facebook^5^, Twitter^5^, and Instagram^6^) to uncover insights about social issues such as health, politics and business. Among these platforms, Twitter stands out as one of the most immediate; tweets flow nonstop on the bulletin boards of users incessantly. Twitter allows users to express and spread opinions, thoughts and emotions as concisely and quickly as possible. Therefore, researchers have often preferred to analyze user comments on Twitter to immediately uncover insights about social issues during the coronavirus pandemic (e.g., conspiracy theories^7^, why people oppose wearing a mask^8^, experiences in health care^9^, and vaccinations^10^) or distance learning^11–13^.

The text content of a tweet is a short microblog message containing at most 280 characters; this feature makes tweets particularly suitable for natural language processing (NLP) techniques, which are widely used to extract insights from unstructured texts. Distance learning was much debated during the pandemic. On the other hand, we chose Twitter for its immediacy in capturing and spreading people’s opinions and emotions on any topic, as well as for its ability to provide plentiful data, even in a short amount of time. Moreover, the people who have more directly experienced distance learning are students, parents, and teachers, that is, people who, by age, make up approximately 83% of Twitter users^46^.

This study aims to explore sentiments and major topics about distance learning in Italy and their evolution over time by using NLP techniques to analyze tweets from Italian Twitter users. Findings from this study could help the Ministry of Education visualize how people are coping with distance learning, thus improving distance learning support and making the experience more effective in the future.

Unlike traditional methods, which are expensive and time-consuming even for small samples, NLP techniques use big data and social media and are very economic, fast, and immediate. A well-known drawback of these methods, however, is that they do not allow us to consider social variables (e.g., age, gender, marital status, mode of working) related to the emotions revealed by the model.

In the literature, COVID-19 has been associated with psychological distress, depression, anxiety, and fear^14–16^. Other research highlights a significant level of traumatic stress in women more than in men^17^. Moreover, pregnant women during lockdowns suffered the most from anxiety and depression^18^.

Regarding age, the research highlights that older people suffered the most from negative effects such as fear and loneliness^19,20^. Younger individuals had fewer negative emotions because they saw COVID-19 as a less risky disease for them^21^, although they did report anxiety and depression due to the social restrictions imposed^21^.

Finally, regarding marital status, Rania and Coppola^22^ show how single, divorced and separated individuals were the most affected by loneliness and demonstrated a higher level of mental illness compared to married individuals. In addition, differences also emerged regarding work during COVID-19. Those who continued to work without changes reported a lower level of mental health than those who switched to working remotely.

## Methodology

### The data

Twitter was chosen as the data source. It is one of the world’s major social media platforms, with 199 million active users in April 2021^4^, and it is also a common source of text for sentiment analyses^23–25^.

To collect distance learning-related tweets, we used TrackMyHashtag https://www.trackmyhashtag.com/, a tracking tool to monitor hashtags in real time. Unlike Twitter API, which does not provide tweets older than three weeks, TrackMyHashtag also provides historical data and filters selections by language and geolocation.

For our study, we chose the Italian words for ‘distance learning’ as the search term and selected March 3, 2020 through November 23, 2021 as the period of interest. Finally, we chose Italian tweets only. A total of 25,100 tweets were collected for this study.

### Data preprocessing

To clean the data and prepare it for sentiment analysis, we applied the following preprocessing steps using NLP techniques implemented with Python:

1. removed mentions, URLs, and hashtags,
2. replaced HTML characters with Unicode equivalent (such as replacing ‘&amp;’ with ‘&’),
3. removed HTML tags (such as $< div>$, $< p>$, etc.),
4. removed unnecessary line breaks,
5. removed special characters and punctuation,
6. removed words that are numbers,
7. converted the Italian tweets’ text into English using the ‘googletrans’ tool.

In the second part an higher quality dataset is required for the topic model. The duplicate tweets were removed, and only the unique tweets were retained. Apart from the general data-cleaning methods, tokenization and lemmatization could enable the model to achieve better performance. The different forms of a word cause misclassification for models. Consequently, the WorldNet library of NLTK^26^ was used to accomplish lemmatization. The stemming algorithms that aggressively reduce words to a common base even if these words actually have different meanings are not considered here. Finally, we lowercased all of the text to ensure that every word appeared in a consistent format and pruned the vocabulary, removing stop words and terms unrelated to the topic, such as ‘as’, ‘from’, and ‘would’.

### Sentiment and emotion analysis

**Figure 1:**
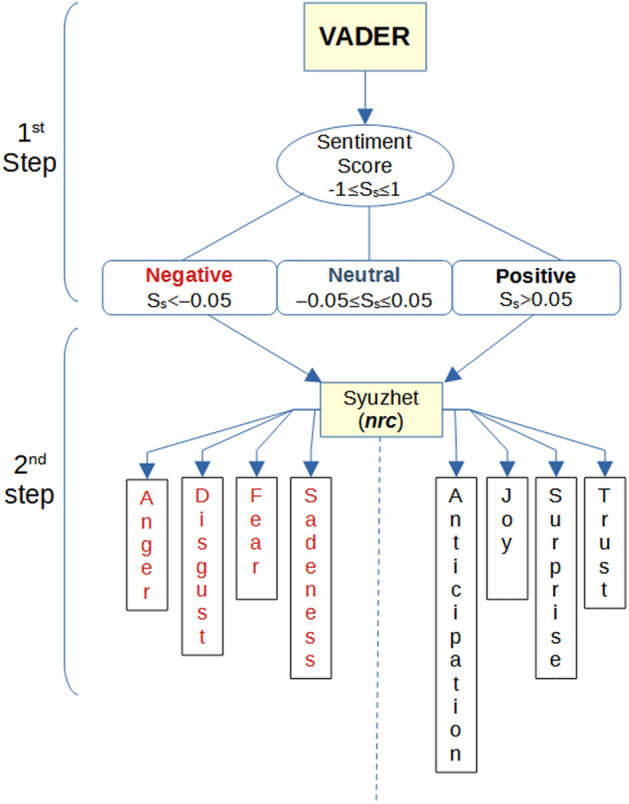
Steps of sentiment and emotion analysis.

Between the major algorithms to be used for text mining and specifically for sentiment analysis, we applied the Valence Aware Dictionary for Sentiment Reasoning (VADER) proposed by Hutto et al.^27^ to determine the polarity and intensity of the tweets. VADER is a sentiment lexicon and rule-based sentiment analysis tool obtained through the wisdom of the crowd approach. Through extensive human work, this tool enables the sentiment analysis of social media to be completed quickly and has a very high accuracy similar to that of human beings. We used VADER to obtain sentiment scores for a tweet’s preprocessed text data. At the same time, according to the classification method recommended by its authors, we mapped the emotional score into three categories: positive, negative, and neutral (Fig. 1 step1).

Then, to discover the emotions underlying categories, we applied the *nrc*^28^ algorithm, which is one of the methods included in the R library package *syuzhet*^29^ for emotion analysis. In particular, the *nrc* algorithm applies an emotion dictionary to score each tweet based on two sentiments (positive or negative) and eight emotions (anger, fear, anticipation, trust, surprise, sadness, joy, and disgust). Emotional recognition aims to identify the emotions that a tweet carries. If a tweet was associated with a particular emotion or sentiment, it scores points that reflect the degree of valence with respect to that category. Otherwise, it would have no score for that category. Therefore, if a tweet contains two words listed in the list of words for the ‘joy’ emotion, the score for that sentence in the joy category will be 2.

When using the *nrc* lexicon, rather than receiving the algebraic score due to positive and negative words, each tweet obtains a score for each emotion category. However, this algorithm fails to properly account for negators. Additionally, it adopts the bag-of-words approach, where the sentiment is based on the individual words occurring in the text, neglecting the role of syntax and grammar. Therefore, the VADER and *nrc* methods are not comparable in terms of the number of tweets and polarity categories. Hence, the idea is to use VADER for sentiment analysis and subsequently to apply *nrc* only to discover positive and negative emotions. The flow chart in Fig. 1 represents the two-step sentiment analysis. VADER’s neutral tweets are very useful in the classification but not interesting for the emotions analysis; therefore, we focused on tweets with positive and negative sentiments. VADER’s performance in the field of social media text is excellent. Based on its complete rules, VADER can carry out a sentiment analysis on various lexical features: punctuation, capitalization, degree modifiers, the contrastive conjunction ‘but’, and negation flipping tri-grams.

### The topic model

The topic model is an unsupervised machine learning method; that is, it is a text mining procedure with which the topics or themes of documents can be identified from a large document corpus^30^. The latent Dirichlet allocation (LDA) model is one of the most popular topic modeling methods; it is a probabilistic model for expressing a corpus based on a three-level hierarchical Bayesian model. The basic idea of LDA is that each document has a topic, and a topic can be defined as a word distribution^31^. Particularly in LDA models, the generation of documents within a corpus follows the following process:

1. A mixture of *k* topics, $\theta$, is sampled from a Dirichlet prior, which is parameterized by $\alpha$;
2. A topic $z_n$ is sampled from the multinomial distribution, $p(\theta \mid \alpha )$ that is the document topic distribution which models $p(z_{n}=i\mid \theta )$ ;
3. Fixed the number of topics $k=1 \ldots ,K$, the distribution of words for *k* topics is denoted by $\phi$ ,which is also a multinomial distribution whose hyper-parameter $\beta$ follows the Dirichlet distribution;
4. Given the topic $z_n$, a word, $w_n$, is then sampled via the multinomial distribution $p(w \mid z_{n};\beta )$.

Overall, the probability of a document (or tweet, in our case) “$\mathbf {w}$” containing words can be described as:

1 $$\begin{aligned} p(\mathbf{w})=\int _\theta {p(\theta \mid \alpha )\left( {\prod \limits _{n = 1}^N {\sum \limits _{z_n = 1}^k {p(w_n \mid z_n ;\beta )p(z_n \mid \theta )} } } \right) } \mathrm{}d\theta \end{aligned}$$

Finally, the probability of the corpus of *M* documents $D=\{\mathbf{w}_\mathbf{1},\ldots ,\mathbf{w}_\mathbf{M}\}$ can be expressed as the product of the marginal probabilities of each single document $D_m$, as shown in (2).

2 $$\begin{aligned} p(D) = \prod \limits _{m = 1}^M {\int _\theta {p(\theta _m \mid \alpha )\left( {\prod \limits _{n = 1}^{N_m } {\sum \limits _{z_n = 1}^k {p(w_{m,n} \mid z_{m,n} ;\beta )p(z_{m,n} \mid \theta _m )} } } \right) } } \mathrm{}d\theta _m \end{aligned}$$

In our analysis that includes tweets over a 2-year period, we find that the tweet content is changeable over time, and therefore, the topic content is not a static corpus. The Dynamic LDA model (DLDA) is adopted and used on topics aggregated in time epochs, and a state-space model handles transitions of the topics from one epoch to another. A Gaussian probabilistic model to obtain the posterior probabilities on the evolving topics along the timeline is added as an additional dimension.

**Figure 2:**
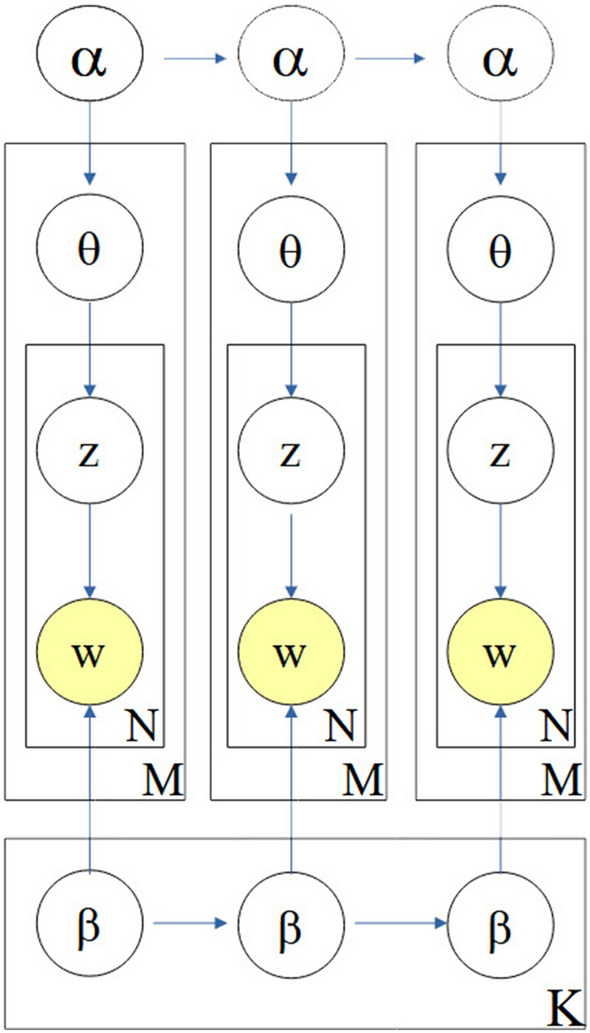
Dynamic topic model (for three time slices). A set of topics in the dataset is evolved from the set of the previous slice. The model for each time slice corresponds to the original LDA process. Additionally, each topic’s parameters evolve over time.

Figure 2 shows a graphical representation of the dynamic topic model (DTM)^32^. As a part of the probabilistic topic model class, the dynamic model can explain how various tweet themes evolve. The tweet dataset corpus used here (March 3, 2020-November 23, 2021) contains 630 days, which is exactly seven quarters of a year. The dynamic topic model is accordingly applied to seven time steps corresponding to the seven trimesters of the dataset. These time slices are put into the model provided by *gensim*^33^.

An essential challenge in DLDA (as LDA) is to determine an appropriate number of topics. Roder et al. proposed coherence scores to evaluate the quality of each topic model. Particularly, topic coherence is the measure used to evaluate the coherence between topics inferred by a model. As coherence measures, we used $C_v$ and $C_{umass}$. The first is a measure based on a sliding window that uses normalized pointwise mutual information (NPMI) and cosine similarity. Instead, $C_{umass}$ is based on document co-occurrence counts, a one-preceding segmentation, and a logarithmic conditional probability as confirmation measure. These values aim to emulate the relative score that a human is likely to assign to a topic and indicate how much the topic words ‘make sense’. These scores infer cohesiveness between ‘top’ words within a given topic. Also considered is the distribution on the primer component analysis (PCA), which can visualize the topic models in a word spatial distribution with two dimensions. A uniform distribution is preferred, which gives a high degree of independence to each topic. The judgment for a good model is a higher coherence and an average distribution on the primer analysis displayed by the pyLDAvis^34^.

## Results

### Sentiment analysis

**Figure 3:**
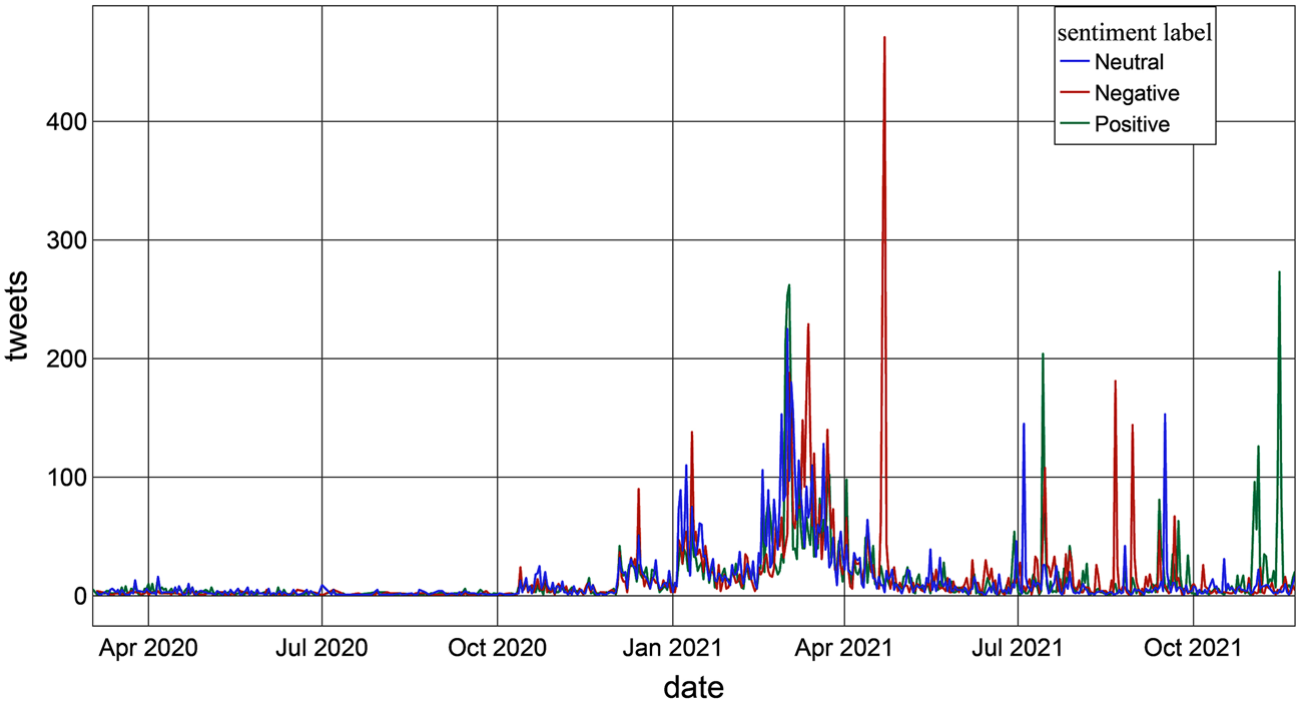
Timeline showing sentiment of tweets about distance learning.

The findings show that the number of tweets has increased since the beginning of distance learning (Fig. 3). Clearly, visible in the graph, there is a significant negative sentiment peak on April 22, 2021, due to the Italian government’s ‘reopening decree’ (DL 2021.4.22 no. 52); it fixed reopenings of schools and commercial activities in gradual terms, depending on the degree of epidemic risk in the different areas.

**Figure 4:**
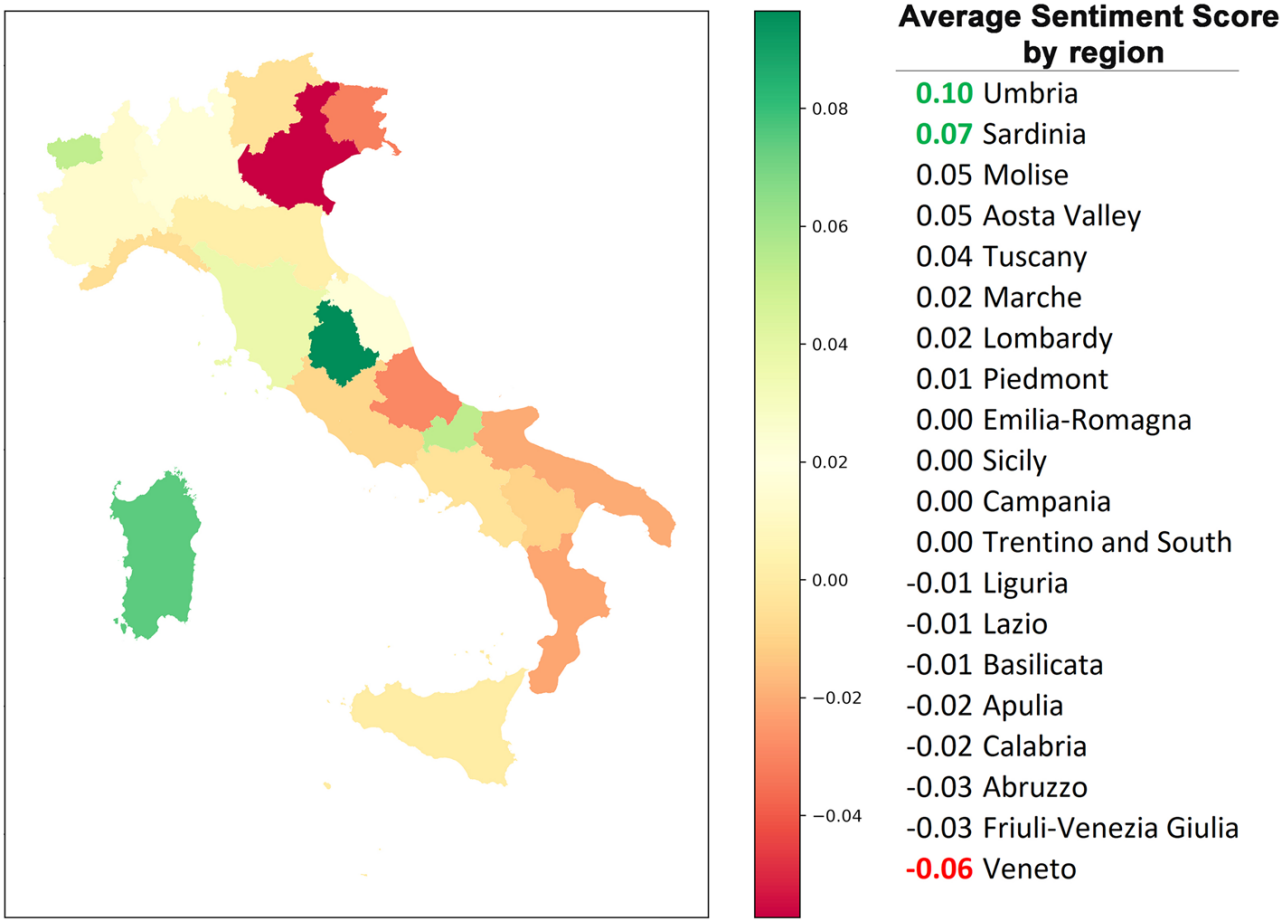
Average Sentiment Score in Italy by region.

Moreover, it is worth noting that the peaks of tweets with a positive sentiment began during the 2021–2022 school year. The highest positive peak was recorded on November 15, triggered by the Italian tax-labor decree draft. Much hyped by the media, it provided for the renewal of extraordinary leave for parents with children involved in distance learning. The output of the VADER model, which is the first step of our sentiment analysis, shows a modest majority of negative tweets: 8843 negative, 8077 neutral and 8180 positive (35.2%, 32.2% and 32.6%, respectively). The analysis carried out at the regional level was performed only on 9534 tweets that had a regional geolocation. Figure 4, shows the average sentiment scores of the Italian regions: the sentiment score is neutral (between − 0.05 and $+$ 0.05, see Fig. 4) for all regions except for Umbria ($+$ 0.10), Sardinia ($+$ 0.07) and Veneto (− 0.06), which slightly exceed the neutrality thresholds. Indeed, there are no major differences in school systems in Italy from region to region. Furthermore, the result is consistent with the flattening due to the use of the average of the scores.

**Figure 5:**
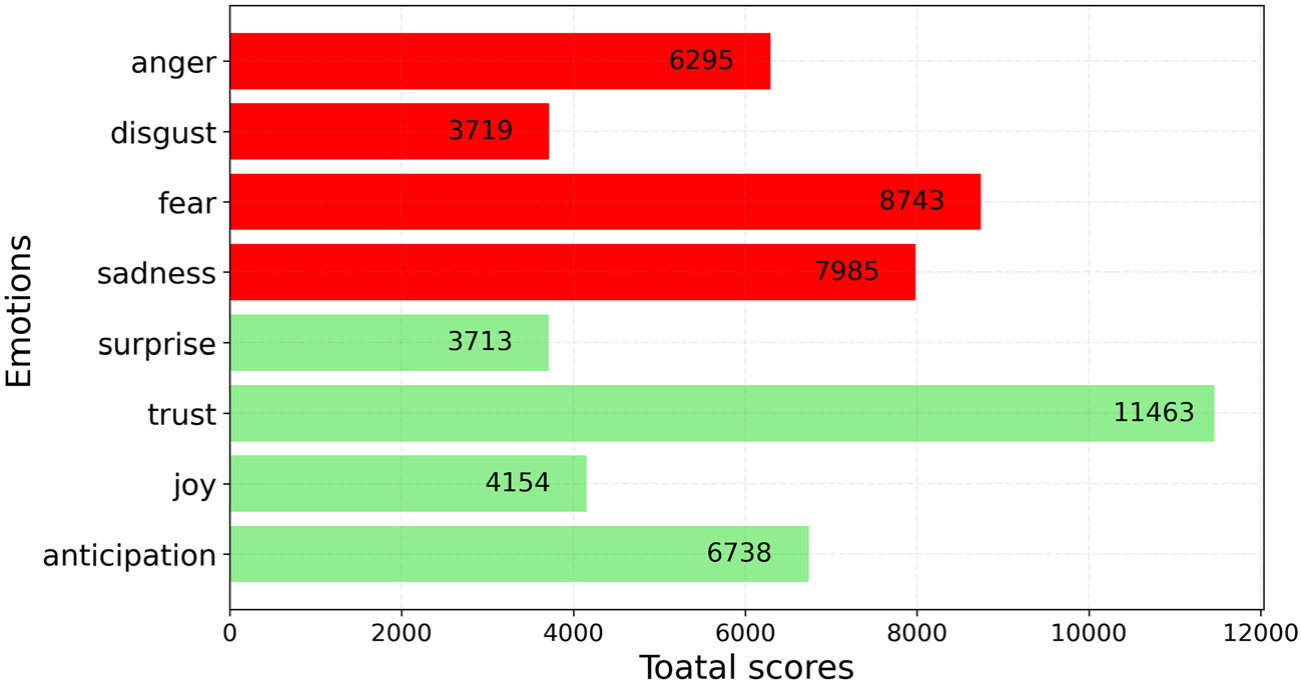
Emotion analysis of non-neutral tweets performed by *syuzhet*.

The second step of the analysis focuses on searching emotions in nonneutral tweets. Among the eight basic emotions, ‘trust’ was the prominent positive emotion observed in the tweets, while ‘fear’, ‘sadness’ and ‘anger’ were the top negative emotions (Fig. 5). These results need to be interpreted in light of recent literature on psychological dimensions of the COVID-19 pandemic. The dimension of fear includes the fear of being infected or infecting others, the risk of death, the loss of loved ones, and not receiving adequate care^35–38^. Several studies performed during the pandemic found that there is an association between fear and depression^14,15,39–41^. Sadness is considered by numerous authors to be a core symptom of depression^42^. The dimensions of anger related to the pandemic include anger at the government and conspiracy mentalities but also anger at those who fail to comply with government hygiene measures to contain the virus^43^.

### The topic model

**Figure 6:**
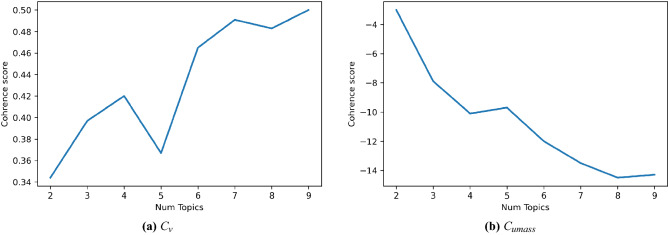
Coherence values.

**Figure 7:**
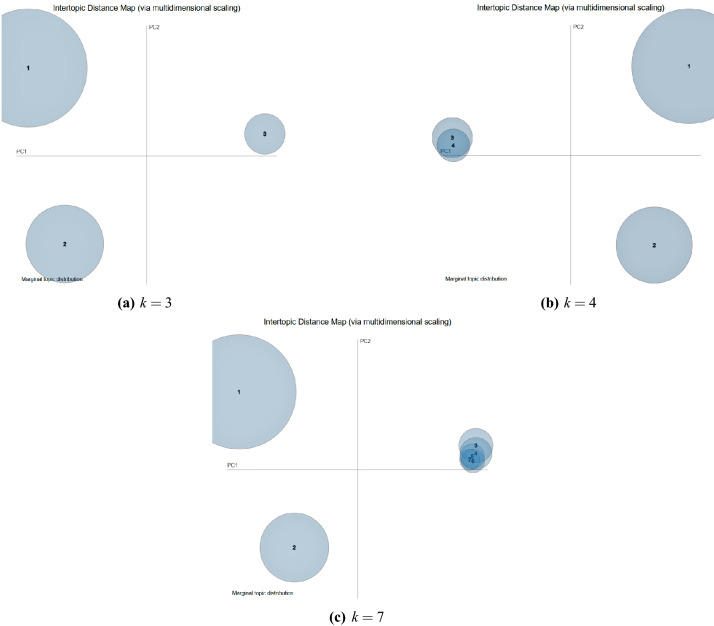
Primer component analysis using average distribution for several topic numbers *k*.

To explore what the user is concerned about on Twitter with reference to distance learning, we applied LDA to our clean corpus. For a better representation of the entire content, it is necessary to find an appropriate topic number. By using topic numbers *k* ranging from 2 to 10, we initialized the LDA models and calculated the model coherence. We mainly used $C_v$ coherence and $C_{umass}$ coherence as a secondary reference. According to Fig. 6, the coherence score peaked at 3, 4, and 7 topics (6 was not considered because $C_{umass}$ did not confirm good coherence for this topic). The choice of 4 or 7 topic numbers would lead to a nonuniform distribution on primer component analysis (PCA), which means that there is not a high degree of independence for each topic. Therefore, we chose 3 as the topic number: the model has no intersections among topics, summarizes the whole word space well, and the topics remain relatively independent (Fig. 7).

**Figure 8:**
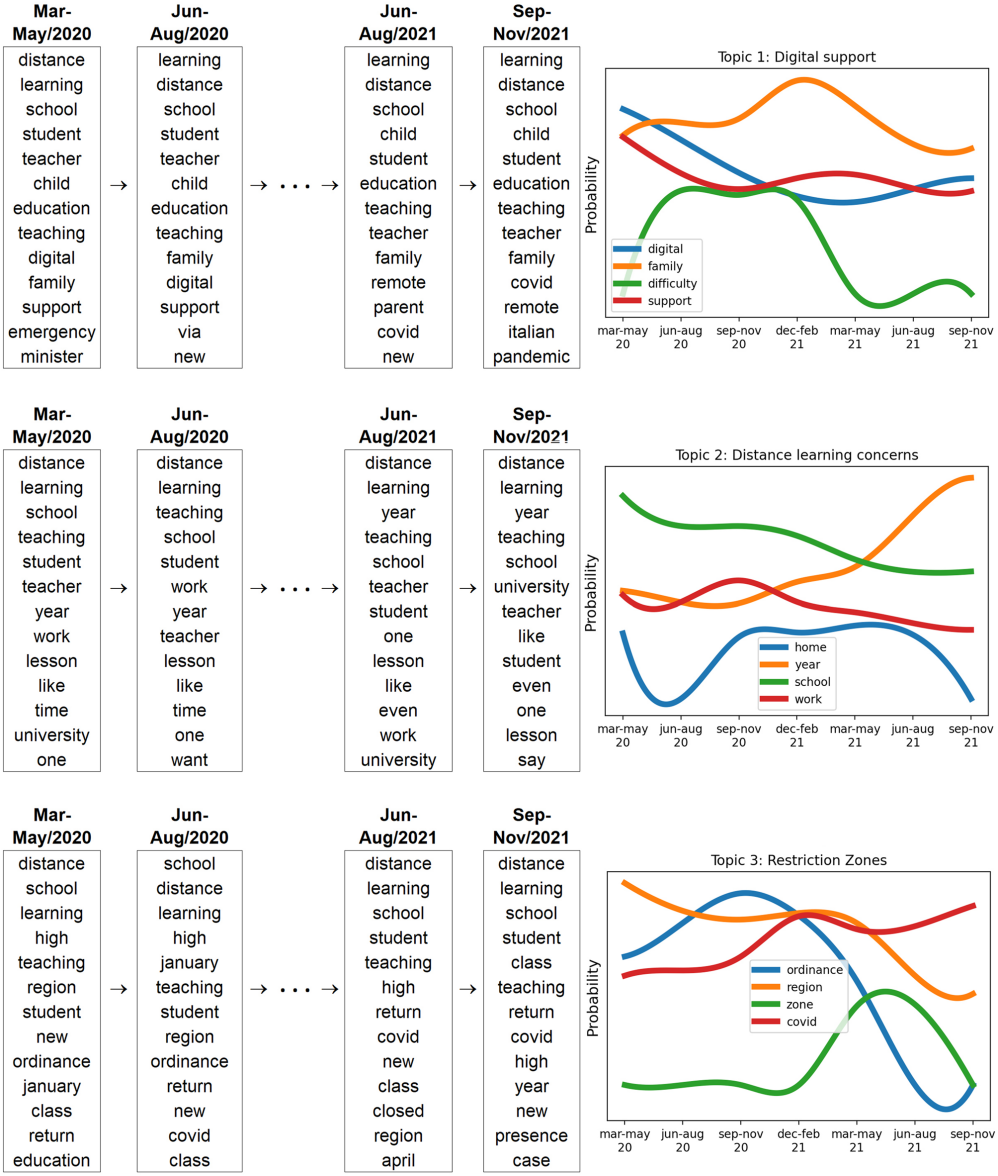
Topics and top terms of different time slices by DTM. Distributions for some relevant terms in each topic over time.

In our analysis, we find that the tweet content changes over time, and therefore, after initializing through the LDA model, its dynamic version (DLDA) is used. Our tweets dataset corpus contains 630 days, which makes exactly seven quarters of a year. The DLDA is accordingly applied to seven time steps corresponding to the seven trimesters of the dataset. The model output (Fig. 8 identified the following three topics:

- Topic 1: Digital support
- Topic 2: Distance learning concerns
- Topic 3: Restriction zones.

The first theme includes words, such as ‘digital,’ ‘family’ and ‘support’, meaning that people need support in distance learning. The second topic includes the words ‘work,’ ‘student,’ and ‘lesson’. Based on this, we inferred that most people complain about social issues and personal problems that are difficult to management due to distance learning. Additionally, several words, such as ‘red,’ ‘zone,’ and ‘ordinance,’ are mentioned in the third topic. This indicates that a further source of anxiety for the Italians was the government decrees introducing the red zones and the corresponding restrictions.

The dynamic topic model shows that the people’s concerns changed over time (Fig. 8). In the topic related to ‘digital support’, the relevance of words such as ‘family’ and ‘support’ remained stable, while the importance of the term ‘difficulty’ decreased in the later stages of the period. Therefore, concerns about support in distance learning were quite stable over time, while difficulties gradually declined.

**Figure 9:**
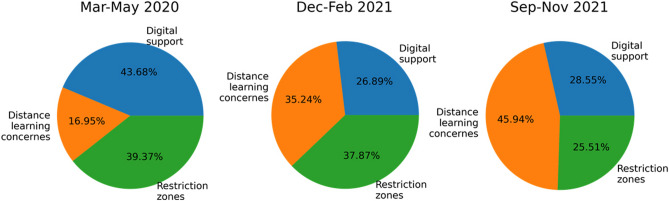
Dynamic volume of the topics over time.

In the topic related to ‘distance learning concerns’, the importance of words such as ‘school’ and ‘work’ remained stable, while the word ‘home’ decreased in importance as time passed until it vanished. These results indicate that concerns about distance learning were stable, but the difficulty of staying home was no longer one of them. Last, in the topic related to ‘restriction zones’, the emphasis on words such as ‘covid’ and ‘region’ remained quite stable, while the term ‘ordinance’ decreased over time. The word ‘zone’, which ranked low at first, started to climb in the middle of the period and went down again. The main finding indicates an increase in concerns about restricted zones, following the Italian government decrees establishing the so-called ‘red zones’, i.e., areas with a high risk of coronavirus infection. The pie charts in Fig. 9 show the dynamic volume of each topic in three periods: March–May 2020, December–February 2021, and September–November 2021. It is worth noting that the fraction of tweets on topic 2 (distance learning concerns) increases considerably from 16.95% in the first period to 45.94% in the last period. On the other hand, the fraction of tweets on topic 1 (digital support) decreased during the second period and then grew slightly in the last period. Finally, the number of tweets on topic 3 (restriction zones) decreased considerably from March 2020 to November 2021.

## Limitations

This study has some limitations. Regarding the emotion analysis, a possible limitation is that the number of emotion categories was limited to 8^28,44^, but emotion is a broad concept and may involve up to 27 categories^45^. Furthermore, misspelled words could not be identified and analyzed in the algorithm. Further limitations concern the dictionary of sentiments (“lexicon”) developed by Mohammad and Turney^28^, which maps a list of language features to emotion intensities:

- Only 5 individuals were recruited to annotate a term against each of the 8 primary emotions.
- The emotions of a term have been annotated without considering the possible contexts.
- Although the percentages of agreement were apparently high, interrater reliability statistics were not reported.

Regarding topic analysis, considering unsupervised learning such as DLDA, the primary limitation is some degree of subjectivity in defining the topic created^10^. Finally, it is worth noting that the most recent statistics about social media usage show that approximately 83% of Twitter users worldwide were under age 50^46^; this implies that Twitter-based studies generally suffer from an underestimation bias in the opinions of people aged 50 and over. However, the distance learning topic truly affects the younger population more closely than the older population; therefore, the underestimation issue may have a marginal, if any, impact on the results in the present study.

## Conclusions and future prospectives

With the aim of studying the opinions and emotions of Italians regarding distance learning, we collected tweets on this issue and carried out a sentiment analysis using the VADER and *syuzhet* packages. The results showed a predominance of negative attitudes. The sentiment analysis shows daily fluctuations (Fig. 3), mainly due to continuous updates by the news media and the succession of government decrees to contain the coronavirus. However, the long-term trend shows an improvement in sentiment until the trend is reversed; attitudes become positive at the beginning of the 2021–22 school year. Of the highest emotions detected, ‘trust’ was found to be the main positive emotion, while ‘fear’, ‘sadness’ and ‘anger’ were the top negative emotions. The topic model identified three topics: (1) requests for support measures for distance learning, (2) concerns about distance learning and its application, and (3) anxiety about the government decrees introducing red zones and corresponding restrictions. What emerges clearly is the change over time in the percentage weight of the topics: the concerns about distance learning assumed an increasing importance to the detriment of the other topics. In the past two years, the use of distance learning has usurped other learning systems due to the pandemic, inducing sudden, dramatic and probably irreversible changes in the education process. The use of digital teaching technologies accelerated and led to a hybrid instructional model that combined remote and face-to-face teaching, named *integrated* digital learning. While distance learning has generated and still generates fears and concerns, integrated digital learning has already proven itself more effective than traditional teaching. The positive peak in time series sentiments started at the beginning of school year 2021–22 (Fig. 3) when *integrated* digital learning was fully applied in Italy. Further, ongoing technological advancements and the growing experience of students and teachers could mitigate any concerns related to a return to distance learning following a new pandemic wave or other crisis. Therefore, future studies could investigate how perceptions and opinions about distance learning will change in the coming years, using sources other than Twitter and combining results of multiple databases.
